# F76. CHILDHOOD TRAUMA RELATED TO ABNORMAL SOCIAL COGNITION IN SCHIZOPHRENIA

**DOI:** 10.1093/schbul/sby017.607

**Published:** 2018-04-01

**Authors:** Aldara Alvarez Astorga, Alba Lubeiro Juárez, Eva Sotelo, Mercedes Vaquero, Vicente Molina Rodriguez

**Affiliations:** 1 University Hospital of Valladolid; 2 University of Valladolid

## Abstract

**Background:**

Childhood trauma has been proposed as a risk factor for schizophrenia. Moreover, it has been related to brain abnormalities associated with cognitive functions, including social cognition. Alterations in mentalizing skills are found in both schizophrenia patients and individuals exposed to childhood trauma. We hypothesize that childhood trauma might be related to emotional processing deficits in psychotic patients.

**Methods:**

The present study is ongoing. To date, we have assessed social cognition and childhood trauma in 30 patients with schizophrenia. Social cognition is quantified using Mayer, Salovey and Caruso emotional intelligence test (MSCEIT) with five different categories: i) emotional perception, ii) emotional facilitation, iii) emotional comprehension, iii) emotional management and iv) emotional intellectual quotient dimensions. Early trauma data is collected using Childhood Trauma Questionnaire (CTQ), which yields physical, emotional, sexual abuse and neglect scores. We have assessed the correlation coefficients (Spearman′s rho) between childhood trauma and social cognition scores.

**Results:**

According to our preliminary analyses, there are significant inverse correlation coefficients in the patients group between emotional neglect and total trauma scores and, on the other hand, social cognition scores for the facilitation, comprehension, management and emotional intellectual quotient dimensions. Thus, patients with higher scores reflecting more severe emotional neglect and total trauma performed lower in social cognition tests.

**Discussion:**

Childhood trauma experiences may contribute to social cognition deficits in schizophrenia.

